# Serum PTH ≥ 40 pg/mL as a Marker of Bone Fragility and Vitamin D Deficiency in Periodontitis Patients: Biochemical, Densitometric and Genetic Evidence

**DOI:** 10.3390/biom15111600

**Published:** 2025-11-14

**Authors:** Giada Marroncini, Serena Martinelli, Francesco Petrelli, Francesco Bombardiere, Antonio Sarnataro, Francesco Saverio Martelli

**Affiliations:** 1 Biomolecular Diagnostic Laboratories, Via N. Porpora, 50144 Florence, Italy; francesco.bombardiere@biomoleculardiagnostic.com (B.F.); antonio.sarnataro@bdmail.it (S.A.); francesco.martelli@ednmail.it (M.F.S.); 2 Department of Clinical and Experimental Medicine, University of Florence, 50139 Florence, Italy; 3 Department of Biology, School of Pharmacy, University of Rome “Tor Vergata”, 00133 Rome, Italy; francesco.petrelli.1@unil.ch; 4 Department of Biomedical Sciences, University of Lausanne, 1005 Lausanne, Switzerland

**Keywords:** parathyroid hormone (PTH), vitamin D (VitD), bone mineral density (BMD), genetic polymorphisms (SNPs)

## Abstract

(1) Background: this study aimed to determine whether a serum parathyroid hormone (PTH) threshold of 40 pg/mL represents a clinically relevant risk factor for vitamin D (VitD) deficiency and reduced bone mineral density (BMD). It also investigated potential genetic interactions influencing PTH regulation and skeletal health in patients with periodontitis. (2) Methods: a cross-sectional analysis was conducted on 1038 periodontitis patients (35–75 years). Serum PTH, VitD, calcium (Ca), phosphate (P), and urinary parameters were assessed. Dual-energy X-ray absorptiometry (DXA) was used to evaluate BMD in 261 subjects. Vitamin D Receptor (VDR) and estrogen receptor alpha (ERα) polymorphisms were genotyped, and composite genetic risk scores were calculated. Statistical analyses included correlation tests, subgroup comparisons, and regression models. (3) Results: sixty-two percent of individuals had PTH > 40 pg/mL, which was associated with significantly lower 25(OH)D and Ca levels and reduced T-scores (*p* < 0.05). PTH levels negatively correlated with BMD (Pearson’s r = –0.159, *p* = 0.0105). Patients with higher ERα polymorphism scores showed increased PTH values (*p* < 0.05), while VDR variants demonstrated a positive but no significant trend. (4) Conclusions: a PTH threshold of 40 pg/mL identifies individuals at higher risk of VitD deficiency and skeletal fragility, even without overt hypercalcemia. Genetic factors, particularly ERα variants, may contribute to elevated PTH levels, suggesting value in integrating biochemical, densitometric, and genetic screening for early bone health risk stratification.

## 1. Introduction

Parathyroid hormone (PTH) is an 84-amino acid polypeptide secreted exclusively by the parathyroid glands. Its release is tightly regulated by circulating levels of ionized calcium (Ca), with hypocalcemia serving as the primary stimulus for increased PTH secretion [1].

PTH is essential for maintaining Ca and phosphate homeostasis and plays a pivotal role in regulating bone mineral density [2]. In the kidneys, PTH enhances tubular reabsorption of Ca, reduces urinary Ca excretion, and stimulates renal 1-alpha-hydroxylase activity, promoting the conversion of 25-hydroxyvitamin D (25(OH)D)to its active form, 1,25-dihydroxyvitamin D (1,25(OH)_2_D) [3]. This active form increases intestinal Ca absorption, contributing to the restoration of serum Ca levels [4].

Physiologically, PTH levels fluctuate in response to serum Ca concentrations [5]. When Ca levels drop, PTH secretion increases to mobilize Ca from the bone, a readily accessible reservoir, into the bloodstream. Once normocalcemia is restored, PTH levels decrease, allowing bone-forming osteoblast activity to resume [4].

VitD also plays a crucial role in maintaining Ca and phosphate balance, partly by regulating the synthesis and secretion of PTH from the parathyroid glands through several coordinated mechanisms. It directly suppresses transcription of the PTH gene by binding to VitD receptors in the parathyroid glands, thereby reducing hormone synthesis [6]. Additionally, VitD increases intestinal Ca absorption, helping maintain normal serum Ca levels, and indirectly inhibits further PTH release through the Ca-sensing receptor [7]. When 25(OH)D levels fall below approximately 50–60 nmol/L, synthesis of 1,25(OH)_2_D decreases, leading to reduced Ca absorption and a compensatory increase in PTH secretion to preserve Ca homeostasis [8].

In the setting of primary hyperparathyroidism (PHPT), PTH secretion becomes dysregulated, leading to elevated hormone levels despite normal or high serum Ca concentrations. This inappropriate secretion is most commonly due to parathyroid adenomas but can also result from familial hypocalciuric hypercalcemia [9]. In contrast, secondary hyperparathyroidism (SHPT) represents an adaptive response to hypocalcemia, often caused by vitamin D (VitD) deficiency, chronic kidney disease, or malabsorption syndromes. In SHPT, elevated PTH levels reflect a compensatory mechanism to increase Ca availability through bone resorption, enhanced renal Ca retention, and stimulation of intestinal absorption [10,11].

Chronic elevation of PTH, whether primary or secondary, exerts significant effects on bone remodeling [12]. PTH directly stimulates osteoclast differentiation and activation via the Receptor Activator of NF-κB Ligand (RANKL) signaling pathway, leading to increased bone resorption [13]. While transient PTH elevations may promote bone formation (an effect leveraged in intermittent PTH analog therapy), sustained elevations favor catabolism over anabolism, resulting in reduced bone mass and compromised bone microarchitecture [11,14]. Consequently, this imbalance contributes to the pathophysiology of osteopenia and osteoporosis, marked by diminished bone strength and increased fracture susceptibility.

Osteoporosis is a chronic, degenerative skeletal disorder defined by decreased bone mass and disruption of bone microarchitecture, increasing the risk of fragility fractures and functional decline [15]. Bone integrity is maintained by continuous remodeling, mediated by osteoblasts, responsible for osteoid synthesis and mineralization, and osteoclasts, which resorb bone matrix. This balance is delicately modulated by systemic hormones such as PTH, VitD, and local cytokines [16].

In individuals with hyperparathyroidism (HPT), the persistent stimulation of osteoclast activity leads to excessive Ca release from the skeleton, often accompanied by hypercalcemia [17,18]. Notably, elevated serum Ca in this context reflects bone demineralization rather than adequate intake or absorption. The resultant structural deterioration underlies the well-established association between HPT and osteoporosis [19]. VitD deficiency frequently coexists with PHPT and SHPT, impairing intestinal Ca absorption and further exacerbating PTH hypersecretion. Additionally, VitD deficiency and elevated PTH levels have been independently associated with increased cardiovascular risk. Mechanistically, high PTH may contribute to hypertension, vascular calcification, smooth muscle dysfunction, and myocardial hypertrophy [20]. PTH has also been implicated in systemic inflammation through suppression of anti-inflammatory cytokines such as interleukin-10 (IL-10), whose levels increase following VitD supplementation [21].

Moreover, the coexistence of low 25(OH)D and elevated PTH has been identified as an independent risk factor for sudden cardiac death in older adults without pre-existing cardiovascular disease [22]. In cases where PTH levels remain elevated without hypercalcemia or an identifiable secondary cause, the condition is termed normocalcemic primary hyperparathyroidism (NPHPT), an increasingly recognized variant that may still confer skeletal and cardiovascular risk [22].

In this study, we demonstrate that a PTH threshold of 40 pg/mL is a significant risk factor for the development of VitD deficiency and the onset of osteoporosis. Elevated PTH levels were inversely correlated with serum 25(OH)D and Ca concentrations, as well as bone mineral density.

## 2. Materials and Methods

### 2.1. Study Population

This cross-sectional study examined 1038 patients who visited a dental clinic between January 2022 and January 2024. The included participants were affected by periodontitis and had available data on serum PTH, 25(OH)D, serum and urinary Ca, or phosphate as well as sex, age, bone mineralization data and instrumental examinations. The main exclusion criteria were (i) the VitD supplementation, (ii) the intake of medication known to affect the concentration of PTH or VitD, including antiepileptic medicine, immune modulating medicine or intake of medically prescribed supplementary PTH, 25(OH)D, Ca, or phosphate. Therefore, the cohort included 1038 participants eligible for analysis, consisting of 437 men and 601 women, aged 35 to 75 years.

The inclusion criteria were: age > 35 and <75 years, male and female participants, ability and willingness to provide written informed consent, ability to comply with study procedures and visits. Participants were required to have stable clinical conditions, no acute illness or hospitalization within the previous four weeks, and preserved renal function (estimated glomerular filtration rate [eGFR] ≥ 60 mL/min/1.73 m^2^). Individuals were also required to have resided in the same geographical area for at least six months to ensure comparable sunlight exposure. Subjects receiving vitamin D or calcium supplementation were included only if on a stable dose for at least three months or if not receiving supplementation during the same period. Patients with hypercalcemic primary hyperparathyroidism, hypoparathyroidism, or chronic kidney disease were excluded. Furthermore, individuals with certain medical conditions or medication histories—such as diagnoses of hypophosphatasia, idiopathic hypocalciuric hypercalcemia, or osteomalacia, and those using bisphosphonates, denosumab, romosozumab, or teriparatide—were also excluded during data retrieval.

The informed consent form for participation to the study was distributed to all participants and signed [23].

### 2.2. Biochemical Tests

For each participant, fasting blood samples were obtained by venipuncture and serum sample were separated the day of clinical examination. PTH was analyzed use CLIA methods (Snibe Diagnostic, Maglumi 800, Shenzhen, China) inter- and intra-assay coefficients of variation 9.28% and 5.74% for low concentrations, and 9.34% and 5.16% for high concentrations. The same method was used for 25(OH)D (Snibe Diagnostic, Maglumi 800, China) with inter- and intra-assay coefficients of variation 6.25% and 3.45% for low concentrations, and 6.13% and 3.17% for high concentrations. Serum and urinary Ca (Ca/S) and phosphate (P/S) were measured by standard automated methods in an BA-220 Automatic Chemistry System autoanalyzer (BioSystem, Barcelona, Spain). All analysis followed standard optimization and quality control as per laboratory policy.

### 2.3. Bone Densitometry

For the Bone mineral density (BMD) analysis, we included 261 subjects and measurements were performed according to the WHO criteria [24]. BMD was assessed using (Dual-energy X-ray absorptiometry) (DXA) measurement. BMD at the lumbar spine (L2–L4) and proximal femur (neck, Ward’s triangle, greater trochanter) was measured by dual-energy X-ray absorptiometry (Dexa).

### 2.4. DNA Genotyping

Patient genomic DNA was isolated from EDTA blood samples or swab using a silica-based, phenol- and chloroform-free process in spin-column and 96-well-plate formats (QIAcube, Qiagen, Hilden, Germany). Each DNA sample was genotyped for Vitamin D Receptor (VDR) ApaI (rs64978 G>T), VDR BsmI (rs63980 G>A), VDR TaqI (rs1056 T>C), VDR FokI (rs30920 T>C), estrogen receptor alpha (ER-alpha) PvII (IVS1-397 T/C) and ER-alpha XbaI (IVS1-351 A/G) using Real Time qPCR (Quant Studio5, ThermoFisher Scientific, Waltham, Massachusetts, USA). Single-Nucleotide Polimorphisms (SNPs) were analyzed using Genotyping Software ( Genotyping, qPCR, version 2020.1.4-Q1-20-build1) (Thermo Fisher Scientific, Waltham, Massachusetts, USA) and archived in the Cloud.

### 2.5. Statistical Analysis

Data analysis was performed by the computer program GraphPad Prism Version 8.4.3 for Windows (GraphPad Software). The statistical significance of value differences was evaluated by Studen’s *t*-test using GraphPad Prism Version 8.4.3 for Windows. A P value of less than 0.05 was considered significant.

Data were expressed as mean ± SE (standard error) or ± SD (standard deviation) as detailed in figure legends. Normally distributed variables, skewed variables, and categorical variables were described using mean ± standard deviation, median (upper quartile, lower quartile), and frequency (percentage), respectively. Serum VitD levels were divided into three groups, deficiency (<20 ng/mL), insufficiency (20–30 ng/mL), and sufficiency (≥30 ng/mL). Comparisons between groups at different VitD levels were made using ANOVA or Kruskal–Wallis tests for continuous variables and chi-square tests for categorical variables. For post hoc tests, we used Bonferroni for multiplicity adjustment. Between-group comparisons of VitD levels between patients with osteopenia and osteoporosis were made using independent samples t-tests. Between-group comparisons of categorical variables between patients with osteopenia and osteoporosis were made using chi-square tests and Fisher exact method. Spearman and Pearson’s correlation analysis were used to test the correlations between serum VitD and PTH levels with lumbar and femoral T-score.

## 3. Results

In our study, 1038 individuals of both sexes and different age groups were analyzed. The mean age of participants was 57.08 ± 10.27 years, with ages ranging from 35 to 75 (57.89 ± 10.07 for females and 57.76 ± 10.5 for males). Vitamin D levels were lower during summer and winter (summer, 38.15 ng/mL (47,3%) and winter, 32.02 ng/mL (52,5%)). The average 25(OH)D levels were 35.1 ng/mL ± 17.9, and the mean PTH level was 51.08 pg/mL ± 27.4, both within the normal reference ranges, as were the Ca and P levels in serum and urine.

Notably, 60% of the individuals had hypovitaminosis (25(OH)D < 30 ng/mL), and 62% had elevated PTH levels (>40 pg/mL). Indeed, other parameters such as Free Triiodothyronine (FT3) and Free Thyroxine (FT4) thyroid hormones, as well as bone alkaline phosphatase (B-ALP), were below the minimum reference range, with values of 2.9 ± 0.5 ng/dL, 5.05 ± 4.8 ng/dL, and 19.2 ± 10.6 U/L, respectively. Lumbar, femoral and feet BMD were assessed and 62.8% of the individuals had T-scores > −1.0, significant for osteopenia (*p* ≤ 0.002 compared with reference ranges) (Table 1).

### 3.1. Baseline Characteristics

#### 3.1.1. Population Analysis for PTH Subgroups

**Figure 1:**
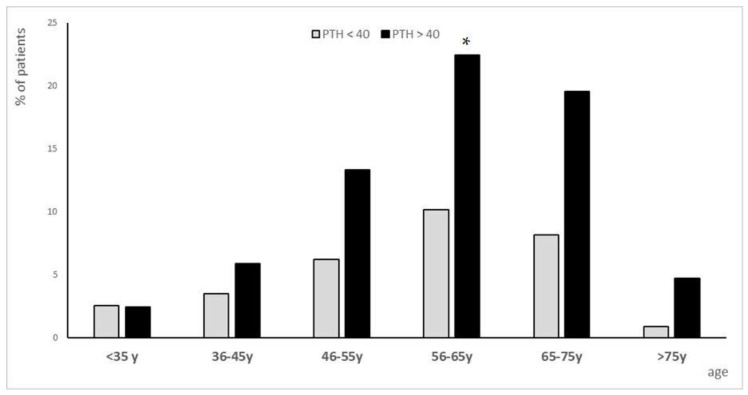
Population subgroups distributed by age. The population (n = 1038) was divided into two groups based on PTH levels < 40 (gray bars) or >40 (black bars). Bar graphs represent the % of individuals clustered by age (<35 years, from 36 to 45 years, from 46 to 55 years, from 56 to 65 years, from 65 to 75 years and >75 years old). * = *p* ≤ 0.05 compared with subgroup with PTH< 40 pg/mL.

The population was divided into two groups according to PTH levels: subgroup1 (<40 pg/mL) and subgroup2 (>40 pg/mL). 62% of individuals had PTH > 40 pg/mL and the distribution based on age showed a statistically significant increase in PTH among 56- and 75-year-olds (Figure 1).

#### 3.1.2. Characterization of Subgroups

**Figure 2:**
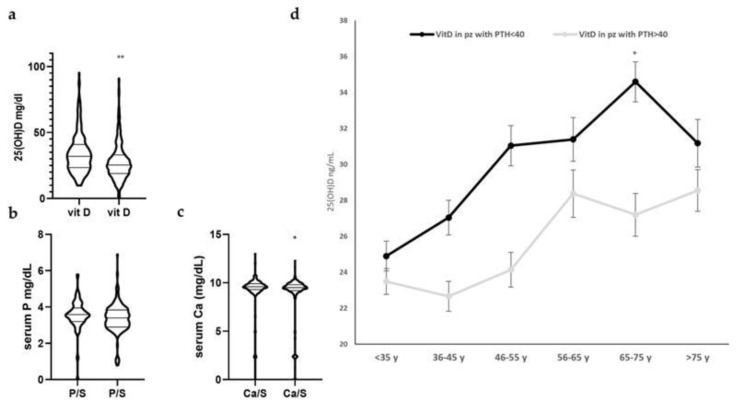
Patients divided according on PTH level of 40 pg/mL showed statistically significant differences on 25(OH)D, serum Ca and P. (**a**–**c**) Scatter dot plots in the subgroup of patients of 25(OH)D, serum P and Ca, respectively. Results are expressed as mean ± SD. (**d**) Graphical representation of 25(OH)D levels in the two subgroups of patients (PTH levels <40 gray line, or ≥40 black line) based on age ranges. (* = *p* < 0.05 and ** = *p* < 0.02 were considered statistically significant).

When individuals were divided by PTH level of 40 pg/mL, the difference between VitD and serum Ca were statistically significant. Therefore, in subgroup1 (PTH < 40 pg/mL) the 25(OH)D mean was 34.22 mg/dL ± 1.026 and in subgroup2 (PTH > 40 pg/mL) was 26.55 mg/dL ± 0.63. Analogously, the serum Ca mean was 9.34 mg/dL ± 0.11 and 8.8 mg/dL ± 0.13, respectively. Based on our results, the PTH value of 40 pg/mL is able to distinguish individuals with SHPT characterized by hypersecretion of PTH, hypovitaminosis D and hypocalcemia (Figure 2).

### 3.2. Bone Mineral Density (BMD)

A total of 261 patients with complete biochemical and densitometric data were included in the BMD analysis. Based on serum PTH concentrations, the cohort was divided into two groups: 146 patients (56%) with high-PTH group (PTH levels > 40 pg/mL) and 115 patients (44%) with normal or low PTH group (PTH levels ≤ 40 pg/mL). The high-PTH group exhibited a significantly higher mean PTH value (62.88 ± 23.56 pg/mL) compared to the low-PTH group (27.75 ± 7.85 pg/mL). Interestingly, the mean 25(OH)D level was also higher in the PTH > 40 group (39.4 ± 24.8 ng/mL) than in those with PTH ≤ 40 pg/mL (32.9 ± 15.5 ng/mL), possibly reflecting or altered hormonal regulation. Of these patients, 33.1% had a normal T-score (T-score > −1), 36.8% had osteopenia (T-score between −1 and −2.5), and 29.8% had osteoporosis (T-score < −2.5) according to WHO guidelines [24,25] (Table 2).

Summary of the main variables in patients with serum parathyroid hormone (PTH) levels higher than 40 pg/mL (n = 146) versus those with PTH ≤ 40 pg/mL (n = 115). Data are presented as mean ± SD. The high PTH group showed significantly elevated PTH concentrations compared to the low PTH group, while their serum VitD levels were slightly, but not significantly, higher. The mean T-score (calculated as the average of lumbar and femoral measurements) was lower in the PTH > 40 group (–1.6 ± 0.8), demonstrating a reduced BMD in this population.

In terms of bone status, the mean T-score (average of lumbar spine and femur) was lower in patients with PTH > 40 pg/mL (–1.6 ± 0.90) than in those with PTH ≤ 40 pg/mL (–1.3 ± 0.90), suggesting greater skeletal involvement in the former group (Table 2).

**Figure 3:**
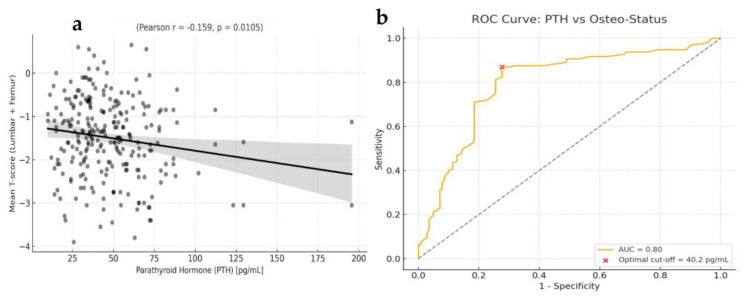
Statistical analysis of serum PTH levels for predicting osteopenia/osteoporosis. (**a**) Correlation between circulating PTH concentrations and the mean T-score (lumbar spine and femur), revealing a significant inverse relationship. (**b**) Receiver operating characteristic (ROC) curve analysis demonstrated good discriminative performance, with an area under the curve (AUC) of 0.797. The optimal threshold, determined using Youden’s index, was 40.2 pg/mL. This cut-off yielded a sensitivity of 87% (95% CI: 81–91%) and a specificity of 72% (95% CI: 64–80%). The red dot indicates the optimal decision point.

A correlation analysis was conducted to explore the relationship between serum PTH levels and the mean T-score. A statistically significant inverse correlation was observed (Pearson’s r = –0.159, *p* = 0.0105; Spearman’s ρ = –0.142, *p* = 0.0228), indicating that higher PTH values are associated with lower BMD. This finding is consistent with the hypothesis that elevated PTH, especially in the context of SHPT, may contribute to bone demineralization, even in patients with normal serum Ca levels (Figure 3a).

A scatterplot with linear regression line shows the relationship between serum PTH concentrations and the mean BMD T-score, calculated as the average of lumbar spine (L1–L4) and total femur values. A statistically significant inverse correlation was observed (Pearson’s r = –0.159, *p* = 0.0105; Spearman’s ρ = –0.142, *p* = 0.0228), indicating that higher PTH levels are associated with lower BMD. The black regression line and confidence interval reflect the linear fit and variability of the data. To further evaluate the diagnostic performance of serum PTH as a continuous variable in predicting osteopenia/osteoporosis, a receiver operating characteristic (ROC) curve analysis was performed. The optimal threshold was determined using Youden’s index, and sensitivity, specificity, and AUC with 95% confidence intervals were reported (Figure 3b).

**Figure 4:**
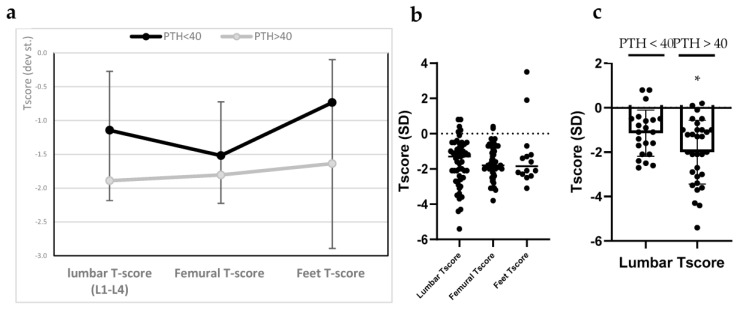
Comparison of T-scores across anatomical sites between patients with PTH ≤ 40 pg/mL and PTH > 40 pg/mL by *t*-test. (**a**) Line graph depicting mean T-scores (±SD) at the lumbar spine (L1–L4), femoral region, and feet in two groups stratified by serum PTH levels. Patients with PTH ≤ 40 pg/mL (black line) exhibited consistently higher T-scores at all measured skeletal sites compared to those with PTH > 40 pg/mL (gray line). The difference was most pronounced in the lumbar spine and foot measurements. (**b**) Bar graph showing the distribution of patients with osteopenia and osteoporosis at each anatomical site (lumbar spine, femur, feet), stratified by PTH levels; (**c**) Box plots illustrating the variation in T-scores across the three anatomical sites in the two PTH groups. Median T-scores were significantly lower in the subgroup of patients with PTH > 40 pg/mL (* = *p* ≤ 0.05 vs. PTH < 40 pg/mL subgroup).

Moreover, Figure 4 shows that patients with PTH ≤ 40 pg/mL consistently have higher T-scores across all measured skeletal regions (lumbar spine (L1–L4), femoral region, and feet) compared to those with PTH > 40 pg/mL. The difference was most pronounced at the lumbar spine (difference statistically significant) and foot levels, where the gap between groups approached or exceeded 0.5 SD.

While femoral T-scores showed the least divergence between the two groups, the overall trend suggests a more pronounced reduction in BMD among individuals with high PTH levels.

### 3.3. Interaction Between Genetic Polymorphisms and PTH Levels

**Figure 5:**
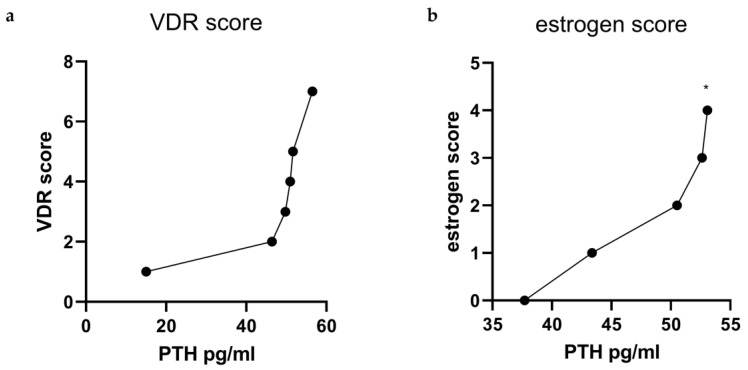
Relationship between cumulative genetic polymorphism burden and PTH levels. Patients were genotyped for six SNPs associated with VitD and estrogenic signaling: (**a**) VDR ApaI (rs64978), BsmI (rs63980), TaqI (rs1056), FokI (rs30920) and (**b**) ERα PvII (IVS1-397 T/C) and XbaI (IVS1-351 A/G). A cumulative polymorphism score was computed by assigning a value of 2 for altered homozygous polymorphic genotypes, 1 for heterozygous, and 0 for wild-type at each locus. The sum of all scores was then plotted against serum PTH levels. The graph shows a positive trend between the number of genetic variants and PTH concentration, with a stepwise increase in mean PTH observed across score categories, both when PTH was plotted against VDR score (**a**) and against ER score (**b**). (* = *p* < 0.05 was considered statistically significant).

To evaluate the potential contribution of genetic predisposition to PTH regulation, we assessed the cumulative burden of polymorphisms within genes involved in VitD signaling and estrogen metabolism. Specifically, we genotyped each individual for four VDR polymorphisms (ApaI (rs64978 G>T), BsmI (rs63980 G>A), TaqI (rs1056 T>C), and FokI (rs30920 T>C)) as well as two ER-alpha polymorphisms (PvII (IVS1-397 T/C) and XbaI (IVS1-351A/G)). To quantify the overall genetic load, we adopted a simple additive scoring model: a score of 2 was assigned for altered homozygous polymorphic genotypes, 1 for heterozygous genotypes, and 0 for wild type. The individual scores across all six single-nucleotide polymorphisms (SNPs) were summed, yielding a composite polymorphism score ranging from 0 to 8 for VDR SNPs and 0 to 4 for ER SNPs. This cumulative score was then correlated with circulating PTH concentrations in the study population. As shown in Figure 5, a positive trend was observed between the genetic polymorphism score and PTH levels, suggesting a potential interaction whereby a higher burden of polymorphic alleles may predispose individuals to elevated PTH levels. The PTH variation among groups reached statistical significance in patients with higher ER SNPs score (*p* < 0.05). Additionally, VDR SNPs scores showed an increase with increasing PTH concentrations, although this trend was not statistically significant. Indeed, the graphical representation demonstrates a consistent increase in PTH values mean with increasing genetic load.

## 4. Discussion

**Figure 6:**
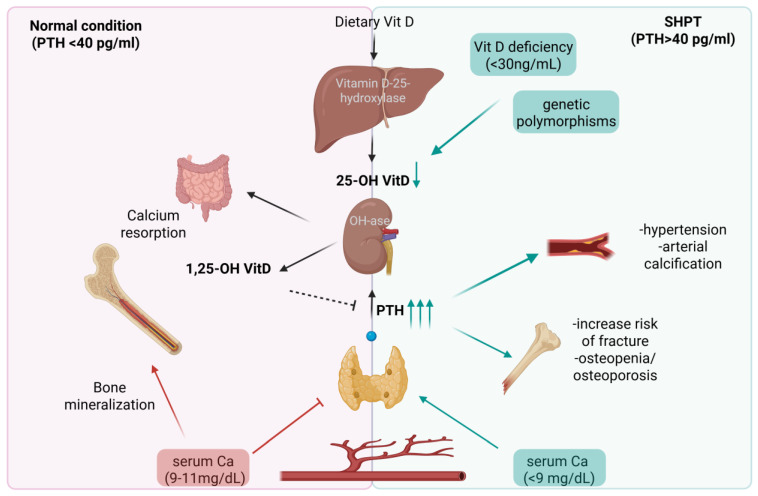
Schematic representation of the physiological differences between normal parathyroid hormone (PTH) levels and secondary hyperparathyroidism (SHPT). On the left, individuals with PTH < 40 pg/mL show normal calcium absorption from the gut, sufficient conversion of vitamin D into its active form (1,25-OH Vit D), proper bone mineralization, and stable serum calcium levels (9–11 mg/dL). On the right, SHPT (PTH > 40 pg/mL) is driven by vitamin D deficiency (<30 ng/mL) and genetic polymorphisms that enhance PTH secretion. This biochemical imbalance leads to reduced serum calcium (<9 mg/dL), increased bone resorption, and diminished bone mineral density, resulting in osteopenia, osteoporosis, and a higher risk of fractures. Moreover, elevated PTH is associated with hypertension and arterial calcification, underscoring its systemic impact. Overall, the illustration highlights the 40 pg/mL PTH threshold as a critical marker of bone fragility and cardiovascular risk, emphasizing the importance of monitoring and managing vitamin D status and PTH levels to maintain skeletal and systemic health. Created with Biorender.

In this study, we demonstrate for the first time that a PTH threshold of 40 pg/mL represents a clinically significant risk factor for both 25(OH)D deficiency and the early onset of osteoporosis. Elevated PTH levels were inversely correlated with serum VitD and Ca concentrations, as well as BMD, highlighting the central role of PTH in skeletal homeostasis. These findings support the concept that sustained elevations in PTH, even in the absence of overt hypercalcemia, may promote bone loss through mechanisms compatible with SHPT, commonly triggered by chronic VitD insufficiency (Figure 6).

Our results are consistent with previous reports identifying elevated PTH as one of the earliest biochemical indicators of VitD depletion, often preceding detectable changes in bone density [26,27,28,29]. By identifying a specific threshold beyond which PTH becomes pathologically relevant, our findings contribute to a more nuanced understanding of the VitD–PTH–bone axis and emphasize the importance of early detection and intervention to mitigate SHPT-related skeletal deterioration.

Although femoral T-scores showed the least divergence between groups, the overall trend indicated a more pronounced reduction in BMD among individuals with elevated PTH levels, reaching statistical significance at the lumbar spine. This is consistent with the known catabolic effects of PTH on trabecular bone, particularly at weight-bearing skeletal sites [30]. Moreover, inclusion of the foot T-score (a site rarely reported in bone densitometry studies) offered additional insight into peripheral skeletal involvement. The lower T-scores observed at this site suggest early skeletal fragility in patients with chronic SHPT and highlight the potential value of evaluating nontraditional sites in assessing systemic bone loss [31]. These data reinforce the systemic impact of elevated PTH on both cortical and trabecular bone, particularly at weight-bearing regions. Importantly, this pattern supports the utility of PTH stratification in clinical bone health assessments and suggests that even borderline elevations in PTH may justify closer monitoring and early therapeutic intervention, particularly when accompanied by decreasing T-scores [32,33].

Importantly, our findings were corroborated by ROC curve analysis, which confirmed the relevance of a PTH threshold around 40 pg/mL in discerning patients with low bone mass. The optimal cut-off of 40.2 pg/mL showed good sensitivity and specificity, supporting the clinical utility of this value for identifying individuals at increased skeletal risk.

Our findings also suggest a genetic component to PTH regulation and its downstream effects on bone metabolism. Specifically, variations in the VDR and ERα genes may modulate individual responses within the Ca–VitD–PTH axis [34]. Patients with higher ERα polymorphism scores exhibited significantly elevated PTH levels, suggesting that genetic variants may influence either PTH secretion or tissue sensitivity. While the trend observed for VDR polymorphisms did not reach statistical significance, the overall direction of the data supports the hypothesis of a gene–environment interaction affecting skeletal outcomes [35,36].

These results are particularly relevant given the composition of our study population, which consisted of individuals with periodontitis, a chronic inflammatory disease that has been previously associated with reduced BMD and systemic bone turnover [37]. Inflammatory mediators and pro-osteoclastogenic cytokines common in periodontitis may exacerbate hormonal and genetic susceptibilities, promoting skeletal fragility. Vitamin D exerts immunomodulatory effects that extend to bone remodeling, partly through the regulation of inflammatory cytokines such as IL-6, TNF-α, and modulation of RANKL expression [38]. Chronic elevations in PTH due to vitamin D deficiency may contribute to bone loss through pro-inflammatory pathways involving Th17 cells, TNF-α, and IL-17 [39]. Furthermore, previous studies have identified associations between periodontal status and VDR gene polymorphisms, reinforcing the complex interplay between immune, hormonal, and genetic factors in bone health [40,41,42,43].

Taken together, these findings support a broader framework in which biochemical, genetic, and inflammatory factors interact to modulate bone integrity. The consistent differences in PTH and T-scores across clinically relevant thresholds suggest that routine measurement of PTH, even in normocalcemic patients, may aid in the early identification of individuals at risk for bone loss. This may be particularly important in populations with coexisting inflammatory conditions or genetic predispositions.

Cyclical variations in PTH levels are expected within the normal physiological range: during the winter months, reduced sunlight exposure leads to lower serum 25(OH)D concentrations, resulting in a compensatory rise in PTH to maintain Ca homeostasis [44]. However, previous research has shown that, although VitD levels vary considerably with seasonal sun exposure, these fluctuations have only minor effects on PTH and Ca levels. In healthy individuals, Ca concentrations remain stable throughout the year, without clinically relevant variations. Therefore, seasonal variations in vitamin D do not influence clinical assessment or decision-making related to PTH and calcium metabolism [45,46].

A limitation of our study is the absence of biochemical and dietary covariates that may influence PTH levels independently of VitD status. Specifically, data on renal function (eGFR), albumin-corrected or ionized Ca, phosphate, magnesium, and dietary Ca intake were not systematically collected or available for the full cohort. These factors are known to modulate PTH secretion and may confound the attribution of elevated PTH levels solely to VitD deficiency-related SHPT. While our findings highlight a robust association between low 25(OH)D and elevated PTH concentrations, the lack of multivariable adjustment for these additional parameters could limit causal inference. Future prospective studies should incorporate these covariates into regression models and consider sensitivity analyses to better isolate the contribution of VitD deficiency to SHPT. Such approaches would further strengthen the mechanistic understanding and clinical interpretation of PTH thresholds in bone health risk stratification. Even more, future studies should aim to validate these findings in larger and more diverse cohorts, preferably through longitudinal designs. Investigating whether targeted interventions along the PTH-VitD axis (especially in genetically predisposed individuals) can prevent or mitigate skeletal complications would be a critical next step. Such research may ultimately inform personalized approaches to bone health, particularly in the context of systemic inflammatory diseases such as periodontitis.

## 5. Conclusions

In conclusion, our study supports a clear link between SHPT, VitD deficiency, and reduced BMD in patients with periodontitis. These findings underscore the need for careful monitoring of VitD and PTH levels in this population, while also highlighting the importance of further exploring the impact of genetic variants on bone metabolism. Integrating biochemical, densitometric, and genetic markers may help identify individuals at higher risk and guide personalized therapeutic approaches.

Moreover, these findings support a broader framework where biochemical, genetic, and skeletal parameters converge to modulate bone health. The high prevalence of periodontitis in this cohort further reinforces the clinical importance of systemic inflammation as a contributing factor in bone fragility. Given the subtle but consistent shifts observed in T-score and PTH across clinically relevant thresholds, routine assessment of PTH, even in normocalcemic patients, may help identify individuals at risk of bone loss earlier.

Future studies should aim to validate these findings longitudinally and assess whether targeted interventions on PTH or VitD pathways, especially in genetically predisposed individuals, can mitigate skeletal complications in inflammatory conditions such as periodontitis.

## Figures and Tables

**Table 1:** Population setting.

Variables	Mean Values	Standard Deviations (SD)	Reference Values
Age/years	61.5	±12.1	
PTH (pg/mL)	51.08	±27.4	10–65 pg/mL
25(OH)D (ng/mL)	35.1	±17.9	>30 ng/mL
Serum Calcium	9.2	±1.7	9–11 mg/dL
Serum Phosphate	3.4	±0.6	2.5–4.5 mg/dL
Urinary Ca/24 h (mg/dL)	222.8	168.5	100–250 mg/24 h
Urinary P/24 h (mg/dL)	492.5	267.5	400–1300 mg/24 h
Homocysteine (µmol/L)	13.4	±6.7	<15 µmol/L
Uric acid (mg/dL)	4.6	±0.6	2.6–7.2 mg/dL
Glycemia (mg/dL)	89.2	±17.6	70–100 mg/dL
Total cholesterol (mg/dL)	188.7	±79.6	<200 mg/dL
LDL (mg/dL)	139.7 *	±53.3	<100 mg/dL
FT3 (pmol/L)	2.9 *	±0.5	3–8 pmol/L
FT4 (pmol/L)	5.05 *	±4.8	9–23 pmol/L
TSH (µIU/mL)	5.2	±9.1	0.4–5.5 µIU/mL
Bone alkaline phosphatase (B-ALP (U/L)	19.2 *	±10.6	50–220 U/L
Lumbar spine (L1-L4) T-score	−1.38 *	±1.56	>−1.0
Femoral neck T-score	−1.65 *	±0.93	>−1.0
Femoral trochanter T-score	−1.36 *	±1.02	>−1.0
Feet T-score	−2.02 *	±0.67	>−1.0

Summary of the characteristics of the patients enrolled in this study (n = 1038). The mean ± SD was reported and compared to the specific reference range; (* = values that deviate from the reference range).

**Table 2:** Clinical and biochemical characteristics of BMD patients stratified by PTH levels.

	PTH > 40	PTH ≤ 40
**N (patients)**	146	115
**PTH (Mean)**	62.8 ± 23.5	27.7 ± 7.8
**25(OH)D (Mean)**	39.3 ± 24.8	32.8 ± 15.4
**T-score Mean (Mean)**	−1.6 ± 0.8	−1.3 ± 0.8

## Data Availability

Data availability access of the database supporting the results and analysis in the article is available by request to the corresponding author. An official letter will be necessary because of institutional requirements.
